# Association between Cardiologist Consultation and Mortality of Stable Patients with Elevated Cardiac Troponin at Admission

**DOI:** 10.3390/diagnostics11122229

**Published:** 2021-11-29

**Authors:** Ah Ran Oh, Jungchan Park, Sooyeon Lee, Kwangmo Yang, Jin-Ho Choi, Kyunga Kim, Joonghyun Ahn, Ji Dong Sung, Seung-Hwa Lee

**Affiliations:** 1 Department of Anesthesiology and Pain Medicine, Samsung Medical Center, Sungkyunkwan University School of Medicine, Seoul 06351, Korea; aran8612@gmail.com (A.R.O.); jc83.park@samsung.com (J.P.); sooyeon1230.lee@samsung.com (S.L.); 2 Center for Health Promotion, Samsung Medical Center, Sungkyunkwan University School of Medicine, Seoul 06351, Korea; kmhi.yang@samsung.com; 3 Department of Emergency Medicine, Samsung Medical Center, Sungkyunkwan University School of Medicine, Seoul 06351, Korea; jhchoimd@gmail.com; 4 Statistics and Data Center, Research Institute for Future Medicine, Samsung Medical Center, Seoul 06351, Korea; kyunga.j.kim@samsung.com (K.K.); jhguy.ahn@samsung.com (J.A.); 5 Department of Digital Health, SAIHST, Sungkyunkwan University, Seoul 06351, Korea; 6 Rehabilitation & Prevention Center, Heart Vascular Stroke Institute, Samsung Medical Center, Sungkyunkwan University School of Medicine, Seoul 06351, Korea; jidong.sung@samsung.com; 7 Department of Biomedical Engineering, National University College of Medicine, Seoul 03080, Korea

**Keywords:** cardiologists, mortality, referral and consultation, troponin

## Abstract

Elevated cardiac troponin (cTn) showed associations with mortality even in stable patients, but management has not been established. We aimed to investigate whether consultation to cardiologists could reduce mortality of stable patients with cTn elevation at admission. We identified 1329 patients with elevated cTn level at hospitalization from outpatient clinic to any department other than cardiology or cardiac surgery between April 2010 and December 2018. The patients were divided into two groups according to cardiologist consultation at admission. For primary outcome, mortality during one year was compared in the crude and propensity-score-matched populations. In 1329 patients, 397 (29.9%) were consulted to cardiologists and 932 (70.1%) were not. Mortality during the first year was significantly lower in patients consulted to cardiologists compared with those who were not (9.8% vs. 14.2%; hazard ratio (HR), 0.50; 95% confidence interval (CI), 0.35–0.72; *p* < 0.001). After propensity-score matching, 324 patients were in the cardiologist consultation group and 560 patients were in the no cardiologist consultation group. One-year mortality was consistently lower in the cardiologist consultation group (10.5% vs. 14.6%; HR, 0.58; 95% CI, 0.39–0.86; *p* = 0.01). Cardiologist consultation may be associated with lower mortality in stable patients with cTn elevation at admission. Further studies are needed to identify effective management strategies for stable patients with elevated cTn.

## 1. Introduction

Laboratory methods for cardiac troponin (cTn) testing have markedly advanced over the past two decades, with lower limits of detection and enhanced assay precision. This has led to cTn serving as an effective tool for clinical decision making in various situations [1,2,3]. In fact, robust evidence indicates the association between elevated cTn level and adverse events [3,4,5,6]. This association was also found in populations without a specific diagnosis [7]. Hence, myocardial injury, defined by elevation of cTn above the 99th percentile upper reference limit, has become an entity in itself, extended from a prerequisite for the diagnosis of myocardial infarction [8].

Previous studies on the clinical use of cTn testing have focused on the use in emergency departments or for cardiovascular and surgical patients [1,3,9,10,11,12]. In these patients, increased cTn levels were associated with high mortality [5,7,13]. In addition, it was reported that myocardial injury detected at admission was related to higher mortalities, regardless of the cause of admission [14,15]. Since an enormous array of clinical conditions, from kidney disease to anemia, may affect the occurrence of myocardial injury, it is anticipated to be higher in patients requiring hospitalization [8]. However, treatment strategy in patients with incidentally diagnosed myocardial injury at admission remains unclear. The role of the cardiologist has demonstrated clinical significance in cardiac care units and for patients with type 2 myocardial infarction [16,17]. In this regard, we evaluated the role of the cardiologist in the treatment of patients with myocardial injury detected at hospitalization for noncardiac cause.

## 2. Materials and Methods

The Institutional Review Board of Samsung Medical Center approved this study and waived the requirement for the written informed consent for access to the registry for this study (SMC 2021-03-165), considering that the registry was curated in deidentified form.

### 2.1. Study Patients

This study used a large, single-center, deidentified cohort consisting of 289,764 consecutive adult patients who were admitted from outpatient clinic to any department other than cardiology or cardiac surgery between April 2010 and December 2018 at the Samsung Medical Center, Seoul, Korea. This cohort was extracted from the institutional electronic archive system using the “Clinical Data Warehouse Darwin-C”, built for investigators to search and retrieve deidentified medical records that contain over 4 million patients with more than 900 million laboratory findings and 200 million prescriptions. This system uses a unique personal identification number for the mortalities apart from our institutional identification, and consistently updates and confirms with the National Population Registry of the Korea National Statistical Office. Baseline characteristics, symptoms, and functional capacity of patients were organized based on admission notes and nursing charts during hospital stay by independent investigators who were blinded to mortality. From this registry, we enrolled the patients with cTn elevated above the 99th percentile upper reference limit at admission by laboratory blood tests. We divided them into those who were consulted by the department of cardiology and those who were not.

### 2.2. Definition & Study Endpoint

At admission, cTn I was measured at the discretion of the attending clinician based on previous medical history and recent symptoms of the patient. To measure cTn I, an automated analyzer (Advia Centaur XP, Siemens Healthcare Diagnostics, Erlangen, Germany) with a highly sensitive immunoassay was used. The lowest limit of detection was 6 ng/L, and the 99th percentile of the upper reference limit was 40 ng/L, as provided by the manufacturer. The comorbidities were curated based on admission notes and administrative data using the International Classification of Diseases-10 codes [18]. Poor functional capacity was defined by the measurement of exercise tolerance before surgery lower than 4 [19]. The primary endpoint was the mortality during the first year, while the 30-day mortality was also compared.

### 2.3. Statistical Analysis

Baseline characteristics of the study patients are presented as the mean ± standard deviation or median with interquartile range (IQR) for continuous variables, and number and percentage for categorical variables. Differences between the groups were compared with the chi-square or Fisher’s exact test for categorical variables and the *t*-test or the Mann–Whitney test for continuous variables, as applicable. Kaplan–Meier curves for mortalities were constructed and compared using the log-rank test. In the crude population, Cox regression analysis with multivariable adjustment was used to compare mortality, and the results were reported as hazard ratio (HR) with 95% confidence interval (CI). Variables that were retained in the multivariable adjustment model were age, sex, admission for scheduled operation, heart failure, diabetes, renal disease, poor functional capacity, and cardiac symptoms. We generated a propensity-score-matched population to further reduce selection bias while maintaining balanced confounding variables between the two groups. For propensity-score matching, we used caliber widths of 1.5 of the pooled standard deviation of the logit of the propensity score on all available variables, and generated 1:2 individually matched populations without replacement. In the matched population, an absolute standard mean deviation of <10% of variables between the groups was deemed a successful balance between the two groups. The study power of analysis based on sample size was calculated, and was 0.83 and 0.99 when the estimated HR was 0.7 and 0.6, respectively [20]. Additionally, we performed subgroup analysis to estimate the interaction between the observed association and variables such as sex, diabetes, admission for scheduled operation, poor functional capacity, and cardiac symptoms. The results of the subgroup analysis are presented in the forest plot. All analyses were performed using R 4.0.2 (Vienna, Austria; Available online http://www.R-project.org/ (accessed on 5 October 2021). Available online: http://www.alz.org/what-is-dementia.asp (accessed on 5 October 2021)). All tests were two-tailed, and *p* < 0.05 was considered statistically significant.

## 3. Results

### 3.1. Baseline Characteristics

From a total of 289,764 patients in the registry, 21,098 (7.3%) had cTn measurement performed at admission. We enrolled 1329 patients with a cTn level elevated above the upper reference limit (0.5% of the entire registry and 6.3% of those with cTn measurement) for this study. The study patients were divided into those who were consulted by the department of cardiology and those who were not: 397 (29.9%) were consulted to cardiologists and 932 (70.1%) were not. The baseline characteristics of the two groups are summarized in Table 1. The median cTn I levels were 86 (IQR 54–177) ng/L in those who were examined by a cardiologist and 94 (IQR 56–238) ng/L in the no cardiologist consultation group (*p* = 0.09). There were more males in the cardiologist consultation group, and they tended to be older and had a higher prevalence of comorbidities such as heart failure, diabetes, and renal disease. The cardiologist consultation group more frequently reported cardiac symptoms such as palpitation, dizziness, and diaphoresis, but they were less likely to be admitted for scheduled surgical procedures. The departments to which the study patients were admitted are summarized in Table S1.

### 3.2. Mortality

**Figure 1:**
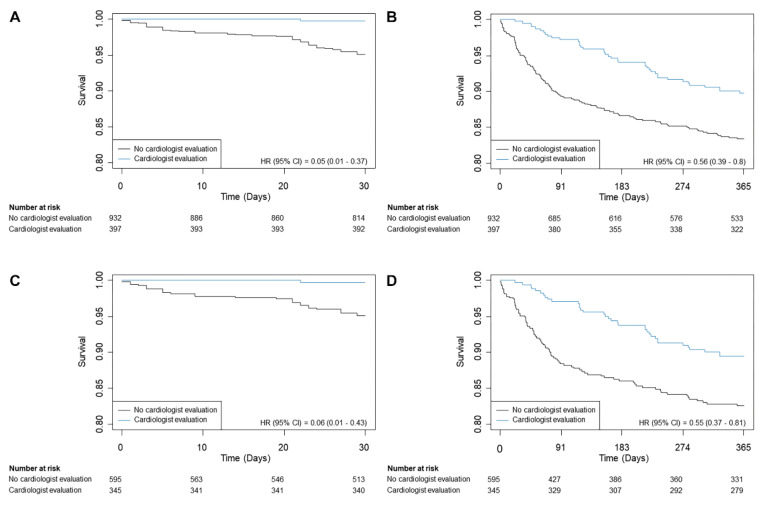
Kaplan–Meier curves of all-cause mortality in the (**A**) 30-day and (**B**) one-year mortalities for the entire population; and (**C**) 30-day and (**D**) one-year mortalities for the propensity-score matched population.

Overall mortality during the first year was 12.9% (171/1329), and the median follow-up duration was 365 days (IQR 365-365). In the crude population, the cardiologist consultation group showed a lower risk of mortality in the first year compared with the no cardiologist consultation group (9.8% vs. 14.2%; HR, 0.50; 95% CI, 0.35–0.72; *p* < 0.001) (Table 2 and Figure 1). The mortality during 30 days was also lower in the cardiologist consultation group (0.3% vs. 4.7%; HR, 0.05; 95% CI, 0.01–0.35; *p* = 0.003).

**Figure 2:**
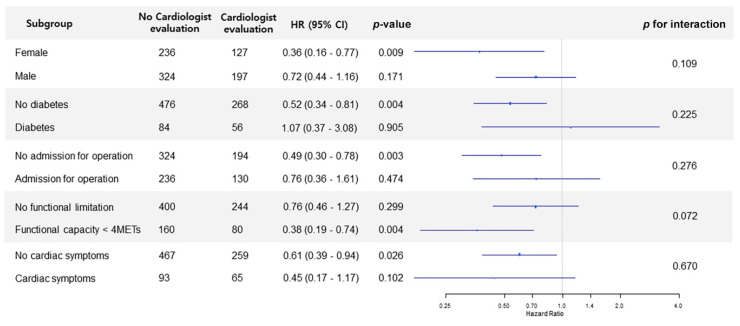
A forest plot of the subgroup analysis.

After propensity-score matching, 324 patients were in the cardiologist consultation group and 560 patients were in the no cardiologist consultation group. The risk of mortality during the first year for the cardiologist consultation group was consistently lower than in the no cardiologist consultation group (10.5% vs. 14.6%; HR, 0.58; 95% CI, 0.39–0.86; *p* = 0.01), and the 30-day mortality was also lower for the cardiologist consultation group (0.3% vs. 4.8%; HR, 0.06; 95% CI, 0.01–0.45; *p* < 0.001) (Figure 1). According to the result of our subgroup analysis, the finding that the cardiologist consultation was associated with the lower one-year mortality did not show significant interaction with any other variable (Figure 2).

### 3.3. Postoperative Management

Cardiologic management during hospital stay and after discharge according to the two groups is presented in Table 3. The median durations for hospital stay were 10 (IQR 6–20) days in the cardiologist consultation group and 11 (IQR 7–22) days in the no cardiologist consultation group. During hospital stay, the patients who were consulted by cardiologists at admission tended to undergo more intensive cardiologic evaluations, including echocardiogram, stress echocardiogram, treadmill test, coronary computed tomographic angiography, and coronary artery angiogram compared to the no cardiologist consultation group. The incidence of myocardial infarction was also higher in the cardiologist consultation group. Accordingly, cardiovascular drugs were more used in the cardiologist consultation group as well as coronary revascularization by percutaneous coronary intervention or coronary artery bypass grafting, but the incidence of intensive care unit treatment was higher in the no cardiologist consultation group

After patient discharge, management showed similar trends. The patients in the cardiologist consultation group underwent more frequent cardiac evaluations, were more often diagnosed with myocardial infarction, and were treated with cardiovascular drugs and coronary revascularization more frequently. Unlike during hospital stay, the incidence of intensive care unit treatment was higher in the cardiologist consultation group.

## 4. Discussion

The current study demonstrated an lower mortality associated with cardiologist consultation in stable patients with cTn elevation at hospitalization. The patients who were consulted by cardiologists were treated with more intensive cardiac evaluation and medical treatment during the in-hospital period and after discharge.

Generally, cTn level is selectively measured in patients with cardiac symptoms such as chest pain or dyspnea, or history of cardiovascular disease. Recent advances in cTn assays and analyzers have extended the use of this cardiac-specified test to various clinical areas such as postoperative evaluation, prognosis prediction after organ transplantation, or even in COVID-19 patients [10,14,21,22]. Despite a concerning perspective for broadening the criteria for cTn measurement, numerous studies have shown the usefulness of cTn test as a diagnostic or prognostic tool in various noncardiac diseases [23,24]. However, adequate management of stable patients with an elevated cTn level remains ambiguous.

In this study, an evaluation by cardiologists was associated with reduced mortality in stable patients with cTn elevation at hospitalization for a noncardiac reason. An association between high-intensity medical staffing and reduced mortality was previously demonstrated, but mostly in the critical care setting [6,16,25,26,27]. Explanations for this association include full attention to patient status and the ability to recognize and manage events promptly [26]. In a recent report on patients with type 2 myocardial infarction, mortality did not differ based on cardiologist evaluation, despite an enormously higher prevalence of risk factors in patients who were evaluated by cardiologists [17]. Considering these inherent differences in risk between the groups, this result can be interpreted as an improvement of outcome by cardiologist evaluation. In our study, the cardiologist evaluation group also had a higher incidence of cardiac risk factors and underwent more frequent cardiac evaluations and therapy including increase of dose and frequency of cardiovascular drug administration that were reported to be beneficial in cardiovascular patients. These management factors were deemed to have affected the lower mortality for the cardiologist evaluation group despite a higher incidence of underlying risk factors and postoperative myocardial infarction.

Additionally, we balanced the difference of cardiovascular risk between the two groups with propensity-score matching. In the propensity-score-matched population, mortality of the cardiology consultation group remained lower with more frequent cardiovascular treatments. The fact that cardiovascular treatment in the cardiologist evaluation group remained higher than in the no cardiologist group even after balancing the differences of variables between the two groups supported our explanation that improved clinical outcomes may be dependent on an appropriate cardiac evaluation and medical treatment. Another possible explanation for our result was that patients with clinically silent myocardial injury at admission might be neglected in the no cardiologist evaluation group. Overall, the results of the current study correlated well with previous studies, and emphasized the role of the cardiologist.

In this study, one-year mortality and 30-day mortality were lower in the cardiologist evaluation group compared to the no cardiologist evaluation group. Generally, ischemic injury is assumed to be an acute event, and hence relevant studies are mostly focused on short-term mortality, rather than long-term outcomes [6,14,27]. So, our result for the long-term follow-up suggested the possibility that improved mortality may not be fully dependent on myocardial injuries that are caused by ischemia. Considering noncardiac origins such as sepsis, chronic kidney disease, stroke, or pulmonary embolism, myocardial injury that is incidentally detected at admission may reflect the poor state of the general condition in some patients, and these patients could be vulnerable to cardiac manifestation. Anticipative cardiac management in these patients may offer improved long-term clinical outcomes.

The results of this study must be interpreted as descriptive, because the data were analyzed using retrospective administrative data. Despite rigorous statistical adjustments with propensity-score matching, unmeasured variables could not be corrected. Secondly, cTn was not a routine evaluation at admission to our institution. It was selectively measured based on discretion of attending clinician, so there is a possibility of selection bias. Moreover, the cardiologist evaluation was also selectively performed at the discretion of the attending clinician, and treatment strategies might have differed between the cardiologists. Therefore, generalization of our results to other populations should be prudent. Despite these limitations, our study showed that the involvement of a cardiologist can reduce mortality in stable patients with cTn elevation at admission.

## 5. Conclusions

Cardiologist consultation in stable patients with cTn elevation at the admission was associated with lower mortality. Further studies are needed to identify effective management strategies for stable patients with elevated cTn.

## Figures and Tables

**Table 1:** Baseline characteristics of the study patients.

	Entire Population				Propensity-Score-Matched Population		
	No Cardiologist Evaluation (*n* = 932)	Cardiologist Evaluation (*n* = 397)	*p*-Value	ASD	No Cardiologist Evaluation (*n* = 595)	Cardiologist Evaluation (*n* = 345)	ASD
Troponin level at admission, ng/L *	94 (56–238)	86 (54–177)	0.09		1357 (±12,248)	2219 (±20,185)	
Age, years	61.7 (±17.3)	65.2 (±13.9)	<0.001	22.1	65.0 (±15.1)	65.0 (±14.4)	0.1
Male	515 (55.3)	256 (64.5)	0.002	18.9	324 (57.9)	197 (60.8)	6
Current smoking	188 (20.2)	86 (21.7)	0.59	3.7	120 (21.4)	72 (22.2)	1.9
Admission for scheduled operation	416 (44.6)	152 (38.3)	0.04	12.9	236 (42.1)	130 (40.1)	4.1
Previous medical history							
Arrhythmia	20 (2.1)	17 (4.3)	0.05	12.1	11 (2.0)	10 (3.1)	7.2
Myocardial infarction	8 (0.9)	7 (1.8)	0.25	8	5 (0.9)	5 (1.5)	5.9
Heart failure	29 (3.1)	33 (8.3)	<0.001	22.6	23 (4.1)	17 (5.2)	5.4
Peripheral vascular disease	4 (0.4)	2 (0.5)	>0.99	1.1	2 (0.4)	1 (0.3)	0.8
Cerebrovascular disease	52 (5.6)	32 (8.1)	0.12	9.9	32 (5.7)	24 (7.4)	6.8
Diabetes	118 (12.7)	98 (24.7)	<0.001	31.2	84 (15.0)	56 (17.3)	6.2
Renal disease	104 (11.2)	109 (27.5)	<0.001	42.2	82 (14.6)	57 (17.6)	8
Poor functional capacity	242 (26.0)	101 (25.4)	0.9	1.2	160 (28.6)	80 (24.7)	8.8
Cardiac symptoms at admission	139 (14.9)	95 (23.9)	<0.001	22.9	93 (16.6)	65 (20.1)	8.9
Chest pain	34 (3.6)	23 (5.8)	0.11	10.1	23 (4.1)	16 (4.9)	4
Palpitation	116 (12.4)	79 (19.9)	0.001	20.3	78 (13.9)	55 (17.0)	8.4
Dizziness	100 (10.7)	70 (17.6)	<0.001	19.9	67 (12.0)	49 (15.1)	9.2
Syncope	7 (0.8)	2 (0.5)	0.89	3.1	6 (1.1)	1 (0.3)	9.2
Diaphoresis	104 (11.2)	79 (19.9)	<0.001	24.3	72 (12.9)	52 (16.0)	9.1
Admission departments							
Departments of medicine	421 (45.2)	182 (45.8)	0.87	1.3	255 (45.5)	142 (43.8)	3.4
Departments of surgery	469 (50.3)	191 (48.1)	0.50	4.4	281 (50.2)	161 (49.7)	1.0
Other departments	42 (4.5)	24 (6.0)	0.30	6.9	24 (4.3)	21 (6.5)	9.7

Data are presented as *n* (%), median (interquartile range), or mean (±standard deviation). Abbreviations: METs, metabolic equivalents; ASD, absolute standardized mean difference. * These variables were not retained in the propensity-score matching.

**Table 2:** Mortalities according to measurement of troponin at admission.

	No Cardiologist Evaluation	Cardiologist Evaluation	Unadjusted HR (CI)	*p*-Value	Adjusted HR (CI)	*p*-Value
Entire population	*n* = 932	*n* = 397				
1-year mortality, No (%)	132 (14.2)	39 (9.8)	0.56 (0.39–0.80)	0.002	0.50 (0.35–0.72)	<0.001
30-day mortality, No (%)	44 (4.7)	1 (0.3)	0.05 (0.01–0.37)	0.003	0.05 (0.01–0.35)	0.003
Propensity-score-matched population	*n* = 560	*n* = 324				
1-year mortality, No (%)	82 (14.6)	34 (10.5)			0.58 (0.39–0.86)	0.01
30-day mortality, No (%)	27 (4.8)	1 (0.3)			0.06 (0.01–0.45)	0.01

Abbreviations: HR, hazard ratio; CI, confidence interval. Multivariable adjustment included age, male, admission for operation, heart failure, diabetes, renal disease, functional capacity, and cardiac symptoms.

**Table 3:** In-hospital and postdischarge management.

	Entire Population			Propensity-Score-Matched Population		
	No Cardiologist Evaluation (*n* = 932)	Cardiologist Evaluation (*n* = 397)	*p*-Value	No Cardiologist Evaluation (*n* = 560)	Cardiologist Evaluation (*n* = 324)	*p*-Value
**Management during hospitalization**						
In-hospital evaluation						
Echocardiogram	419 (45.0)	220 (55.4)	0.001	257 (45.9)	171 (52.8)	0.06
Stress echocardiogram	9 (1.0)	9 (2.3)	0.11	8 (1.4)	6 (1.9)	0.84
Treadmill test	4 (0.4)	1 (0.3)	>0.99	2 (0.4)	1 (0.3)	>0.99
Coronary computed tomographic angiography	8 (0.9)	4 (1.0)	>0.99	7 (1.2)	3 (0.9)	0.91
Coronary artery angiogram	85 (9.1)	66 (16.6)	<0.001	56 (10.0)	50 (15.4)	0.02
In-hospital diagnosis						
Myocardial infarction	16 (1.7)	14 (3.5)	0.07	10 (1.8)	10 (3.1)	0.31
ST-elevation	1 (0.1)	0	>0.99	1 (0.2)	0	>0.99
Non ST-elevation	15 (1.6)	14 (3.5)	0.05	9 (1.6)	10 (3.1)	0.22
In-hospital cardiovascular drugs						
Beta-blocker	142 (15.2)	83 (20.9)	0.02	91 (16.2)	70 (21.6)	0.06
Calcium channel blocker	322 (34.5)	182 (45.8)	<0.001	212 (37.9)	135 (41.7)	0.30
Statin	254 (27.3)	159 (40.1)	<0.001	175 (31.2)	115 (35.5)	0.22
Warfarin	72 (7.7)	56 (14.1)	<0.001	50 (8.9)	42 (13.0)	0.08
Antiplatelet	415 (44.5)	234 (58.9)	<0.001	273 (48.8)	175 (54.0)	0.15
Renin angiotensin aldosterone system inhibitor	356 (38.2)	211 (53.1)	<0.001	240 (42.9)	169 (52.2)	0.01
Direct oral anticoagulant	57 (6.1)	20 (5.0)	0.52	37 (6.6)	18 (5.6)	0.63
In-hospital care						
Percutaneous coronary intervention	20 (2.1)	22 (5.5)	0.002	12 (2.1)	13 (4.0)	0.16
Coronary artery bypass grafting	6 (0.6)	5 (1.3)	0.42	6 (1.1)	4 (1.2)	>0.99
Intensive care unit	502 (53.9)	187 (47.1)	0.03	286 (51.1)	148 (45.7)	0.14
ECMO	55 (5.9)	24 (6.0)	>0.99	27 (4.8)	17 (5.2)	0.91
Continuous renal replacement therapy	23 (2.5)	13 (3.3)	0.52	11 (2.0)	7 (2.2)	>0.99
Ventilator	67 (7.2)	14 (3.5)	0.02	35 (6.2)	13 (4.0)	0.21
**Management after discharge**						
Postdischarge evaluation						
Echocardiogram	170 (18.2)	336 (84.6)	<0.001	114 (20.4)	268 (82.7)	<0.001
Stress echocardiogram	6 (0.6)	14 (3.5)	<0.001	4 (0.7)	11 (3.4)	0.01
Treadmill test	28 (3.0)	16 (4.0)	0.43	13 (2.3)	13 (4.0)	0.22
Coronary computed tomographic angiography	13 (1.4)	21 (5.3)	<0.001	10 (1.8)	18 (5.6)	0.004
Coronary artery angiogram	18 (1.9)	87 (21.9)	<0.001	14 (2.5)	62 (19.1)	<0.001
Postdischarge diagnosis						
Myocardial infarction	5 (0.5)	14 (3.5)	<0.001	3 (0.5)	7 (2.2)	0.06
ST-elevation	2 (0.2)	2 (0.5)	0.74	1 (0.2)	2 (0.6)	0.63
Non ST-elevation	3 (0.3)	12 (3.0)	<0.001	2 (0.4)	5 (1.5)	0.13
Postdischarge cardiovascular drugs						
Beta-blocker	137 (14.7)	148 (37.3)	<0.001	81 (14.5)	128 (39.5)	<0.001
Calcium channel blocker	272 (29.2)	237 (59.7)	<0.001	181 (32.3)	182 (56.2)	<0.001
Statin	294 (31.5)	236 (59.4)	<0.001	191 (34.1)	181 (55.9)	<0.001
Warfarin	62 (6.7)	89 (22.4)	<0.001	45 (8.0)	67 (20.7)	<0.001
Antiplatelet	349 (37.4)	284 (71.5)	<0.001	241 (43.0)	220 (67.9)	<0.001
Renin angiotensin aldosterone system inhibitor	317 (34.0)	277 (69.8)	<0.001	221 (39.5)	217 (67.0)	<0.001
Direct oral anticoagulant	63 (6.8)	69 (17.4)	<0.001	41 (7.3)	57 (17.6)	<0.001
Postdischarge care						
Percutaneous coronary intervention	12 (1.3)	36 (9.1)	<0.001	9 (1.6)	21 (6.5)	<0.001
Coronary artery bypass grafting	0	4 (1.0)	0.01	0	2 (0.6)	0.26
Intensive care unit	76 (8.2)	176 (44.3)	<0.001	50 (8.9)	140 (43.2)	<0.001
ECMO	6 (0.6)	15 (3.8)	<0.001	6 (1.1)	14 (4.3)	0.004
Continuous renal replacement therapy	6 (0.6)	20 (5.0	<0.001	5 (0.9)	14 (4.3)	0.002
Ventilator	6 (0.6)	22 (5.5)	<0.001	4 (0.7)	17 (5.2)	<0.001

Abbreviation: ECMO, extracorporeal membranous oxygenation.

## Data Availability

The data presented in this study are available on request from the corresponding author.

## Supplementary Materials

The following are available online at https://www.mdpi.com/article/10.3390/diagnostics11122229/s1, Table S1: Department of hospitalization.
