# Supplementary material for: Association between Cardiologist Consultation and Mortality of Stable Patients with Elevated Cardiac Troponin at Admission
Source: Diagnostics (Basel). 2021 Nov 29;11(12):2229. doi: 10.3390/diagnostics11122229 (PMC8700380; doi:10.3390/diagnostics11122229)
Supplement: Supplementary file 1 [file diagnostics-11-02229-s001.zip › diagnostics-1490541-supplementary.pdf]

Supplemental Table S1. Department of hospitalization

	Entire population		Propensity-score matched population	
	No cardiologist evaluation (N=932)	Cardiologist evaluation (N=397)	No cardiologist evaluation (N=560)	Cardiologist evaluation (N=324)
Dentist	2 (0.2)	1 (0.3)	1 (0.2)	1 (0.3)
Otolaryngology	11 (1.2)	10 (2.5)	6 (1.1)	9 (2.8)
Family medicine	1 (0.1)	0	1 (0.2)	0
General surgery	246 (26.4)	117 (29.5)	152 (27.1)	90 (27.8)
Gastroenterology	39 (4.2)	19 (4.8)	21 (3.8)	18 (5.6)
Pulmonology	119 (12.8)	42 (10.6)	69 (12.3)	38 (11.7)
Endocrinology	8 (0.9)	9 (2.3)	6 (1.1)	5 (1.5)
Nephrology	63 (6.8)	47 (11.8)	42 (7.5)	22 (6.8)
Hemato-oncology	78 (8.4)	41 (10.3)	53 (9.5)	38 (11.7)
Infectious disease	35 (3.8)	10 (2.5)	19 (3.4)	9 (2.8)
Allergy	6 (0.6)	3 (0.8)	2 (0.4)	3 (0.9)
Rheumatology	6 (0.6)	1 (0.3)	5 (0.9)	1 (0.3)
Neurology	35 (3.8)	7 (1.8)	24 (4.3)	5 (1.5)
Neurosurgery	80 (8.6)	11 (2.8)	38 (6.8)	11 (3.4)
Obstetrics and Gynecology	22 (2.4)	3 (0.8)	9 (1.6)	3 (0.9)
Ophthalmology	1 (0.1)	3 (0.8)	1 (0.2)	3 (0.9)
Orthopedics	75 (8.0)	24 (6.0)	51 (9.1)	23 (7.1)
Pediatrics	30 (3.2)	2 (0.5)	13 (2.3)	2 (0.6)
Plastic surgery	8 (0.9)	0	6 (1.1)	0
Psychiatry	7 (0.8)	2 (0.5)	4 (0.7)	2 (0.6)
Rehabilitation Medicine	33 (3.5)	21 (5.3)	19 (3.4)	18 (5.6)
Health care center	0	1 (0.3)	0	1 (0.3)
Urology	26 (2.8)	23 (5.8)	18 (3.2)	22 (6.8)
